# Editorial for Special Issue “Microorganisms and Organic Waste Valorisation”

**DOI:** 10.3390/microorganisms10081493

**Published:** 2022-07-25

**Authors:** Camilla M. Braguglia, Simona Rossetti

**Affiliations:** Water Research Institute, National Research Council of Italy (IRSA-CNR), Via Salaria, km 29.300, 00185 Roma, Italy

Increasing amounts of organic waste are produced globally from a wide range of industrial activities, wastewater treatment plants, agricultural processing, and human food consumption. Currently, a great amount of attention is being paid to the biorefinery concept based on the sustainable conversion of such waste streams to high-value products and energy. One of the most significant challenges in the field relates to the possibility of controlling microbial pathways to promote favourable metabolic processes, which will in turn increase product specificity and yields.

The seven articles included in this Special Issue provide an insight into the latest findings in organic waste valorisation based on biological processes driven by specialised pure or mixed microbial cultures.

Two articles shed light on lactate-driven chain elongation mechanisms by applying isolation techniques and metagenomics assessment [1,2] in order to promote the production of medium-chain fatty acids with high economic market value such as caproate.

Alias et al. [3] propose urban waste solid-state fermentation as a new eco-designed process that can be used to obtain useful bio-products such as citric acid to extract metals for waste recycling. Tonanzi et al. [4] report an innovative approach based on the anaerobic hydrolysis of waste activated sludge to recover embedded polymeric substances and metals.

One contribution [5] evaluates digestate valorisation through fungal solid-state fermentation to produce an ameliorated biofertiliser as a value-added product. Brémond et al. [6] evaluate the efficacy of digestate post-treatment with isolated ligninolytic aerobic consortia in increasing methane recovery during recirculation. A contribution evaluating the influence of different operational conditions on bacterial communities enriched in phototrophic mixed culture systems appropriately designed for PHA production [7] concludes this series of publications.

Given these diverse contributions, it is evident that organic waste valorisation processes driven by microorganisms will continue to prosper, contributing to a more circular bioeconomy. The main challenge will be the development of biotechnological solutions for sustainable, applicable, and environmentally friendly waste processing methods with a multidisciplinary approach.

## References

[1] Liu B., Popp D., Müller N., Sträuber H., Harms H., Kleinsteuber S. (2020). Three Novel *Clostridia* Isolates Produce *n*-Caproate and *iso*-Butyrate from Lactate: Comparative Genomics of Chain-Elongating Bacteria. Microorganisms.

[2] Crognale S., Braguglia C.M., Gallipoli A., Gianico A., Rossetti S., Montecchio D. (2021). Direct Conversion of Food Waste Extract into Caproate: Metagenomics Assessment of Chain Elongation Process. Microorganisms.

[3] Alias C., Bulgari D., Bilo F., Borgese L., Gianoncelli A., Ribaudo G., Gobbi E., Alessandri I. (2021). Food Waste-Assisted Metal Extraction from Printed Circuit Boards: The *Aspergillus niger* Route. Microorganisms.

[4] Tonanzi B., Gallipoli A., Gianico A., Annesini M.C., Braguglia C.M. (2021). Insights into the Anaerobic Hydrolysis Process for Extracting Embedded EPS and Metals from Activated Sludge. Microorganisms.

[5] Alias C., Bulgari D., Gobbi E. (2022). It Works! Organic-Waste-Assisted *Trichoderma* spp. Solid-State Fermentation on Agricultural Digestate. Microorganisms.

[6] Brémond U., Bertrandias A., Hamelin J., Milferstedt K., Bru-Adan V., Steyer J.-P., Bernet N., Carrere H. (2022). Screening and Application of Ligninolytic Microbial Consortia to Enhance Aerobic Degradation of Solid Digestate. Microorganisms.

[7] Almeida J.R., Fradinho J.C., Carvalho G., Oehmen A., Reis M.A.M. (2022). Dynamics of Microbial Communities in Phototrophic Polyhydroxyalkanoate Accumulating Cultures. Microorganisms.

